# Isolation and Identification of *Candida* from the Oral Cavity

**DOI:** 10.5402/2011/487921

**Published:** 2011-10-25

**Authors:** Smitha Byadarahally Raju, Shashanka Rajappa

**Affiliations:** ^1^Department of Oral Pathology & Microbiology, Sri Hasanamba Dental College & Hospital, Karnataka, Hassan 573201, India; ^2^Department of General Surgery, Hassan Institute of Medical Sciences, Karnataka, Hassan 573201, India

## Abstract

Various techniques are available for the isolation of *Candida* within the oral cavity. Such methods play an important role in the diagnosis and management of oral candidosis. The growing importance of *Candida* is in part related to the emergence of HIV infection and the more widespread use of immunosuppressive chemotherapy. Along with the *Candida albicans* there has been a greater recognition of the importance of the nonalbicans *Candida* species in oral candidosis. Identification of infecting strains of *Candida* is important because isolates of *Candida* species differ widely, both in their ability to cause infection and also in their susceptibility to antifungal agents. Thus this review provides an overview of the reliable methods of candidal isolation and identification of isolates from the oral cavity.

## 1. Introduction

The term *Candida *originates from the Latin word candid, meaning white. The spores of *Candida* are a commensal, harmless form of a dimorphic fungus that becomes invasive and pathogenic pseudohyphae when there is a disturbance in the balance of flora or in debilitation of the host [1]. 

 The translation of this endogenous commensal to the disease-causing parasite may be associated with factors other than the pathogenic attributes of the organism itself, which is rather unique compared with most of the other infectious diseases, where the virulence of the organisms considered being the key factor in the pathogenesis. Hence *Candida* species are strictly opportunistic. It could be stated with that neither the superficial nor the systemic forms of *Candida* infections could be initiated in the absence of underlying pathology [2]. 

There are many species of *Candida* (Table 1), [3] but the most prevalent one which is recovered from the oral cavity, in both commensal state and in cases of oral candidosis, is *C. albicans. *It is estimated that this species accounts for over 80% of all oral yeast isolates.

In recent years there has been an increased interest in infections caused by the opportunistic pathogen *Candida*. The growing importance of *Candida* is in part related to the emergence of HIV infection and the more widespread use of immunosuppressive chemotherapy [4, 5]. Identification of infecting strains of *Candida* is important because isolates of *Candida* species differ widely, both in their ability to cause infection [6] and also in their susceptibility to antifungal agents [7]. 

Along with the *C. albicans* there has been a greater recognition of the importance of the non-albicans *Candida *species in human disease. *C. glabrata *and *C. krusei *are species that have received attention due to their enhanced resistance to certain antifungal agents. *C. dubliniensis *is a recently identified pathogenic species, first described in 1995 when it was coisolated with *C. albicans *from cases of oral candidosis in HIV infected individuals [8].

This review provides an overview of the reliable methods of candidal isolation and identification of isolates from the oral cavity.

## 2. Pathogenic Attributes of *Candida*

The transition of *Candida *from a harmless commensal to a pathogenic organism is complex and is related to subtle environmental changes that lead to expression of a range of virulence factors (Table 2). It is the combined effect of both host and candidal factors that ultimately contribute to the development of oral candidosis [8].

Regardless of the type of candidosis, the ability of *Candida *species to persist on mucosal surfaces of healthy individuals is an important factor contributing to its virulence. This is particularly important in the oral cavity, where the organism has to resist the mechanical washing action of a relatively constant flow of saliva toward the esophagus [9].

No single predominant virulence factor for *Candida *is recognized although there are a number of factors that have been implicated in promoting the infection process. These include attributes involved in the adhesion of *Candida *to oral surfaces (e.g., relative cell surface hydrophobicity and the presence of specific adhesin molecules), the ability to resist host immune defence mechanisms (e.g., high frequency phenotypic switching and morphological transition), and the release of hydrolytic enzymes (e.g., secreted aspartyl proteinases and phospholipases) that can induce damage to host cells [8].

## 3. Oral Candidosis 

Samaranayake [10] proposed a classification where the oral candidosis lesions were subdivided into two main groups: Group I, or primary oral candidoses confined to lesions localized to the oral cavity with no involvement of skin or other mucosae; Group II or secondary oral candidoses, where the lesions are present in the oral as well as extraoral sites such as skin (Table 3). Group I lesions consist of the classic triad—pseudomembranous, erythematous, and hyperplastic variants—and some have suggested further subdivision of the latter into plaque-like and nodular types [11].

## 4. Diagnosis of Oral Candidosis

Diagnosis of oral candidosis can often be made on the nature of the clinical presenting features although microbiological specimens should be taken if possible in order to both identify and quantify any *Candida* that may be present and provide isolates for antifungal sensitivity testing.

## 5. Methods of Isolation

Techniques available for the isolation of *Candida* within the oral cavity include the use of a smear, a plain swab [8], an imprint culture [12], collection of whole saliva [13], the concentrated oral rinse [14], and mucosal biopsy. Each method has particular advantages and disadvantages and the choice of sampling technique is primarily governed by the nature of the lesion to be investigated (Table 4). Where an accessible and defined lesion is evident, a direct sampling approach such as the use of a swab or an imprint is often preferred as this will provide information of the organisms present at the lesion itself. In cases where there are no obvious lesions or in instances where the lesion is difficult to access, an indirect sample based on culturing saliva specimens or an oral rinse is more acceptable. 

Quantitative estimation of fungal load can be done using imprints, concentrated oral rinse, and culturing of oral rinse, as a means of differentiating between commensal carriage and pathogenic existence of oral *Candida*, with higher loads considered likely in the latter [8].

## 6. Direct Microscopy

Morphological features of *Candida* species [15] (Table 5) need to be examined for identification. A smear is of value in differentiating between yeast and hyphal forms but is less sensitive than cultural methods [16]. Potassium hydroxide (KOH) preparation of the specimen reveals nonpigmented septate hyphae with characteristic dichotomous branching (at an angle of approximately 45°) [17]. In KOH-Calcofluor fluorescent-stain method fungal characteristics like hyphae, yeast cells, and other fungal elements will fluoresce [18]. 

A smear taken from the lesional site is fixed on to microscope slides and then stained either by the gram stain or by the periodic acid Schiff (PAS) technique. Using these methods, candidal hyphae and yeasts appear either dark blue (Gram-stain) or red/purple (PAS) [19]. 

In case of chronic hyperplastic candidosis, a biopsy of the lesion is necessary for subsequent detection of invading *Candida *by histological staining using either the PAS or Gomori's methenamine silver stains. Demonstration of fungal elements within tissues is done as they are dyed deeply by these stains. The presence of blastospores and hyphae or pseudohyphae may enable the histopathologist to identify the fungus as a species of *Candida *and, given the presence of other histopathological features, make a diagnosis of chronic hyperplastic candidosis [20].

## 7. Laboratory Culture

### 7.1. Swab

A swab of a lesional site is a relatively simple method of detecting growth and semiquantitative estimation of *Candida* can be obtained. The sampling approach involves gently rubbing a sterile cotton swab over the lesional tissue and then subsequently inoculating a primary isolation medium such as Sabouraud's dextrose agar (SDA) [21].

### 7.2. Concentrated Oral Rinse

The oral rinse technique involves the patient holding 10 mL of sterile phosphate-buffered saline (0.01 M, pH 7.2) in the mouth for 1 minute. The solution is then concentrated (10-fold) by centrifugation and a known volume, usually 50 *μ*L, inoculated on an agar medium using a spiral plating system. After 24–48 hrs incubation at 37°C, growth is assessed by enumeration of colonies and expressed as candidal colony forming units per mL (cfu mL^−1^) of rinse [16].

### 7.3. Imprint Culture

The imprint method utilises a sterile foam pad of known size (typically 2.5 cm^2^), previously dipped in an appropriate liquid medium, such as Sabouraud's broth, immediately before use. The pad is then placed on the target site (mucosa or intraoral prosthesis) for 30 seconds and then transferred to an agar for culture [16].

## 8. Culture Media

The most frequently used primary isolation medium for *Candida* is SDA [22] which, although permitting growth of *Candida*, suppresses the growth of many species of oral bacteria due to its low pH. Incorporation of antibiotics into SDA will further increase its selectivity [8]. Typically SDA is incubated aerobically at 37°C for 24–48 hrs. *Candida* develops as cream, smooth, pasty convex colonies on SDA and differentiation between species is rarely possible [17]. It is estimated that more than one *Candida* species occurs in approximately 10% of oral samples and in recent years the ability to detect nonalbicans species has become increasingly important [16]. 

 In recent years, other differential media have been developed that allow identification of certain *Candida *species based on colony appearance and colour following primary culture. The advantage of such media is that the presence of multiple *Candida* species in a single infection can be determined which can be important in selecting subsequent treatment options [8]. Examples of these include Pagano-Levin agar or commercially available chromogenic agars, namely, CHROMagar *Candida*, Albicans ID, Fluroplate, or Candichrom albicans [16].

Pagano-Levin agar distinguishes between *Candida *species based on reduction of triphenyltetrazolium chloride. The medium produces pale-coloured colonies of *C. albicans*, whilst colonies of other *Candida *species exhibit varying degrees of pink coloration. Pagano-Levin agar has a similar sensitivity to SDA but is superior for the detection of more than one species in the sample [23], CHROMagar *Candida* identifies *C. albicans, C. tropicalis, *and *C. krusei* based on colony colour and appearance [24], whilst Albicans ID and Fluroplate have proven beneficial for the presumptive identification of *C. albicans *[25]. The specificity of identification is reported to be 95% for CHROMagar *Candida* [26] and 98.6% for Albicans ID and Fluroplate agars [25]. The use of CHROMagar *Candida* as a primary isolation agar has been cited as an approach that permits discrimination of the newly described *C. dubliniensis *[27] from *C. albicans*. On CHROMagar *Candida*, *C. dubliniensis *reportedly develops as darker green colonies compared with those of *C. albicans *[28]. However, discrimination between these two species using CHROMagar appears to decline upon subculture and storage of isolates. Failure of *C. dubliniensis *to grow on agar media at the elevated incubation temperature of 45°C has recently been suggested as an alternative test to discriminate between these two species [29]. 

## 9. Identification of *Candida* Species

Identification of yeasts based on primary culture media can be confirmed through a variety of supplemental tests traditionally based on morphological (Table 5) and physiological characteristics of the isolates. 

### 9.1. Morphological Criteria

The germ-tube test is the standard laboratory method for identifying *C. albicans*. The test involves the induction of hyphal outgrowths (germ tubes) when subcultured in horse serum at 37°C for 2–4 hours. Approximately 95% of *C. albicans *isolates produce germ tubes, a property also shared by *C. stellatoidea *and *C. dubliniensis* [16].

*C. albicans *and *C. dubliniensis *can also be identified from other species based on their ability to produce morphological features known as chlamydospores. Chlamydospores are refractile, spherical structures generated at the termini of hyphae following culture of isolates on a nutritionally poor medium such as cornmeal agar. Isolates are inoculated in a cross hatch pattern on the agar and overlaid with a sterile coverslip. Agars are incubated for 24–48 hours at 37°C and then examined microscopically for chlamydospore presence [8]. 

### 9.2. Physiological Criteria/Biochemical Identification

Biochemical identification of *Candida* species is largely based on carbohydrate utilization. Traditional testing would have involved culture of test isolates on a basal agar lacking a carbon source. Carbohydrate solutions would then be placed within wells of the seeded agar or upon filter paper discs located on the agar surface. Growth in the vicinity of the carbon source would indicate utilization. Commercial systems are based on the same principle but test carbohydrates are housed in plastic wells located on a test strip. Growth in each well is read by changes in turbidity or colour changes in certain kit systems. Numerical codes obtained from the test results are used to identify the test organism based on database comparison [30].

### 9.3. Serology

Serological tests are frequently used to ascertain the clinical significance of *Candida* species isolates. Rising titers of lgG antibodies to *C. albicans* may reflect invasive candidiasis in immunocompetent individuals. The detection of IgA and IgM antibodies is important to identify an acute infection. Immunosuppressed individuals often show variability in antibody production and in such a case the use of an antigen detection test is recommended. Tests like enzyme linked immunosorbent assay (ELISA) and radio immuno assay (RIA) for detection of candidial antigen, either cell-wall mannan or cytoplasmic constituents are now available in developed countries [31].

Serological diagnosis is often delayed and the tests still lack sensitivity and specificity. Furthermore, antibody production in immunocompromised patients is variable, making diagnosis complicated [32]. This is due to the fact that fungal antigens and metabolites are often cleared rapidly from the circulation and the presence of antibodies does not always imply a *Candida* infection, especially in patients with serious underlying disease or who are taking immunosuppressive drugs [33].

Serologic tests are normally not a diagnostic tool for oral candidosis. However, such tests may be a prognostic instrument in patients with severe oral candidosis who respond poorly to antimycotic therapy [34].

### 9.4. Molecular-Based Identification Methods

Identification by analysis of genetic variability is a more stable approach than using methods based on phenotypic criteria. For the identification of *Candida* based on genetic variation are analyses of electrophoretic karyotype differences and restriction fragment length polymorphisms (RFLPs) using gel electrophoresis or DNA-DNA hybridization [16].

 Species-specific PCR approaches have also been used for *Candida* species identification. Several target genes have been reported for *Candida *species discrimination, although those most frequently amplified are the sequences of the ribosomal RNA operon. Identification can be obtained based on PCR product sizes obtained following gel electrophoresis resolution, or PCR product sequence variation determined either by direct sequencing or through the use of restriction fragment analysis following cutting of PCR sequences with restriction endonucleases [8].

Fluorescence in situ hybridization with peptide nucleic acid method (PNA Fish) is a new detection technique which targets highly conserved species-specific sequences in the abundant rRNA of living *C. albicans*. Individual cells can be detected directly without the need for amplification [35]. This technique achieves a sensitivity of 98.7–100%, with a specificity of 100%, allowing for the discrimination of *C. albicans* from the phenotypically similar *C. dubliniensis* [36]. 

Molecular-based technology can also be used to identify strains of *Candida *species although the use of techniques such as Pulsed Field Gel Electrophoresis (PFGE), Random Amplified Polymorphic DNA (RAPD) analysis, and repeat sequence amplification PCR (REP) are largely reserved for epidemiological investigations in research of oral candidosis [8].

## 10. Conclusion

**Figure 1:**
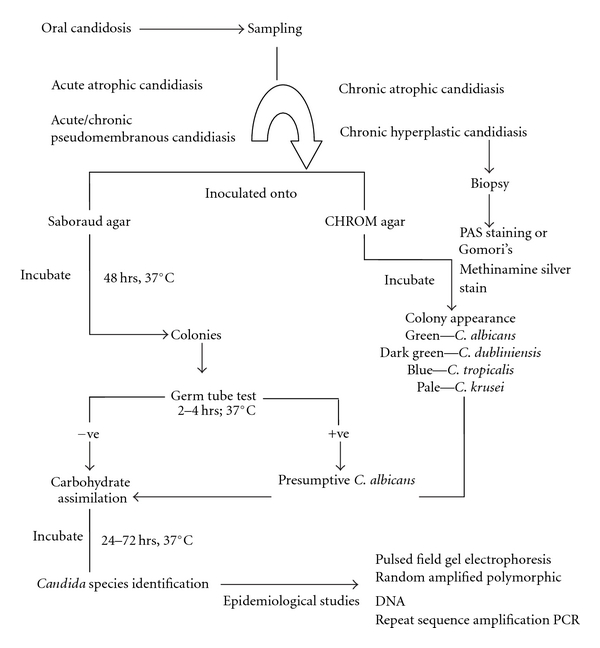
Schematic representation of isolation and identification of *Candida* species from the oral cavity.

In recent years a greater emphasis has been given for reliable identification of *Candida* species from human clinical samples. A schematic representation for candidal isolation and identification is presented in Figure 1. Since *Candida* is the resident microflora, appropriate isolation methods are required to ascertain the presence in the mouth along with their number. It is also important to identify the infecting strains of *Candida* because isolates of *Candida* species differ widely, both in their ability to cause infection and also in their susceptibility to antifungal agents. Various phenotypic techniques are available for identifying isolated *Candida* including using morphological culture tests, differential agar media, and biochemical assimilation tests. These methods are supplemented with recent molecular techniques largely reserved for epidemiological investigations.

## Figures and Tables

**Table 1:** Species of *Candida*.

*Candida albicans*
*Candida glabrata *
*Candida dubliniensis*
*Candida guilliermondii *
*Candida krusei*
*Candida lusitaniae*
*Candida parapsilosis*
*Candida tropicalis*
*Candida kefyr *

**Table 2:** Virulence factors associated with *Candida Albicans*.

Virulence factor	Effect
*Adherence*	*Promotes retention in the mouth *
Relative cell surface hydrophobicity	Nonspecific adherence process
Expression of cell surface adhesion molecules	Facilitates specific adherence mechanisms
*Evasion of host defenses*	*Promotes retention in the mouth*
High frequency phenotypic switching	Antigenic modification through frequent cell surface changes
Hyphal development	Reduces likelihood of phagocytosis; allows phagocytosed yeast to escape phagocyte
Secreted aspartyl proteinase production	Secretary IgA destruction
Binding of complement molecules	Antigenic masking
*Invasion and destruction of host tissue*	*Enhances pathogenicity *
Hyphal development	Promotes invasion of oral epithelium
Secreted aspartyl proteinase production	Host cell and extracellular matrix damage
Phospholipase production	Damage to host cells

**Table 3:** Classification of oral candidosis.

Primary oral candidosis (Group I)	Secondary oral candidosis (Group II)	
The “primary triad”:	Condition	Subgroup
Pseudomembranous (mainly acute)	Familial chronic mucocutaneous candidosis	1
Erythematous (acute/chronic)	Diffuse chronic mucocutaneous candidosis	2
Hyperplastic (mainly chronic)	Candidosis endocrinopathy syndrome	3
(i) Plaque-like	Familial mucocutaneous candidosis	4
(ii) Nodular/speckled	Severe combined immunodeficiency	5a
*Candida-associated lesions*	Di George syndrome	5b
Denture stomatitis	Chronic granulomatous disease	5c
Angular cheilitis	Acquired immunodeficiency syndrome	6
Median rhomboid glossitis	—	—
Linear gingival erythema	—	—

**Table 4:** Methods of recovering *Candida* from the oral cavity.

Isolation method	Advantages	Disadvantages
Culture of whole saliva	Sensitive; viable organisms isolated	Problems may occur with collection of sample; not site specific
Concentrated oral rinse	Quantitative; viable cells isolated	Some patients have difficulty in using rinse; not site specific
Swab	Simple to use; viable cells isolated; site specific	Difficult to standardize
Smear	Simple to use; not reliant on culture	Viable cells not determined; species identity not readily confirmed
Imprint culture	Quantitative; viable cells isolated; site specific	Some sites difficult to sample
Biopsy	Essential for chronic hyperplastic candidosis	Invasive; not appropriate for other forms of candidosis

**Table 5:** Morphological features of *Candida* species.

Feature	
Size (*μ*m)	3–6
Shape	Spherical or oval
Number of buds	Single; chains
Attachment of buds	Narrow
Thickness	Thin
Pseudohyphae &/or hyphae	Characteristic
Number of nuclei	Single

## References

[1] Zunt SL (2000). Oral candidiasis: diagnosis and treartment. *The Journal of Practical Hygiene*.

[2] Soysa NS, Samaranayake LP, Ellepola ANB (2008). Antimicrobials as a contributory factor in oral candidosis—a brief overview. *Oral Diseases*.

[3] Scully C, Ei-Kabir M, Samaranayake LP (1994). *Candida* and oral candidosis: a review. *Critical Reviews in Oral Biology and Medicine*.

[4] Epstein JB, Komiyama K, Duncan D (1986). Oral topical steroids and secondary oral candidiasis. *Journal of Oral Medicine*.

[5] Pomerantz S, Sarosi GA (1992). Fungal diseases in AIDS. *Current Opinion in Infectious Diseases*.

[6] Allen CM, Saffer A, Meister RK, Beck FM, Bradway S (1994). Comparison of a lesion-inducing isolate and a non-lesional isolate of *Candida albicans* in an immunosuppressed rat model of oral candidiasis. *Journal of Oral Pathology and Medicine*.

[7] McIlroy MA (1991). Failure of fluconazole to suppress fungemia in a patient with fever, neutropenia and typhlitis. *Journal of Infectious Diseases*.

[8] Marsh PD, Martin M (2009). Oral fungal infections. *Oral Microbiology*.

[9] Sitheeque MAM, Samaranayake LP (2003). Chronic hyperplastic candidosis/candidiasis (candidal leukoplakia). *Critical Reviews in Oral Biology and Medicine*.

[10] Samaranayake LP, Scully C (1991). Superficial fungal infections. *Current Opinions in Dentistry*.

[11] Holmstrup P, Bessermann M (1983). Clinical, therapeutic, and pathogenic aspects of chronic oral multifocal candidiasis. *Oral Surgery Oral Medicine and Oral Pathology*.

[12] Davenport JC (1970). The oral distribution of *Candida* in denture stomatitis. *The British Dental Journal*.

[13] Oliver DE, Shillitoe EJ (1984). Effects of smoking on the prevalence and intraoral distribution of *Candida albicans*. *Journal of Oral Pathology*.

[14] Samaranayake LP, MacFarlane TW, Lamey P-J, Ferguson MM (1986). A comparison of oral rinse and imprint sampling techniques for the detection of yeast, coliform and Staphylococcus aureus carriage in the oral cavity. *Journal of Oral Pathology*.

[15] Okungbowa FI, Dede APO, Isikhuemhen OS (2009). Cell morphology variations and budding patterns in *Candida* isolates. *Advances in Natural and Applied Sciences*.

[16] Williams DW, Lewis MAO (2000). Isolation and identification of *Candida* from the oral cavity. *Oral Diseases*.

[17] Baveja C (2010). Medical mycology. *Text Book of Microbiology for Dental Students*.

[18] Harrington BJ, Hageage GJ (1991). Calcofluor white: tips for improving its use. *Clinical Microbiology Newsletter*.

[19] Davenport JC, Wilton JMA (1971). Incidence of immediate and delayed hypersensitivity to *Candida albicans* in denture stomatitis. *Journal of Dental Research*.

[20] Nassar A, Zapata M, Little JV, Siddiqui MT (2006). Utility of reflex gomori methenamine silver staining for Pneumocystis jirovecii on bronchoalveolar lavage cytologic specimens: a review. *Diagnostic Cytopathology*.

[21] Axéll T, Simonsson T, Birkhed D, Rosenborg J, Edwardsson S (1985). Evaluation of a simplified diagnostic aid (Oricult-N) for detection of oral candidoses. *Scandinavian Journal of Dental Research*.

[22] Odds FC (1991). Sabouraud('s) agar. *Journal of Medical and Veterinary Mycology*.

[23] Samaranayake LP, MacFarlane TW, Williamson MI (1987). Comparison of Sabouraud dextrose and Pagano-Levin agar media for detection and isolation of yeasts from oral samples. *Journal of Clinical Microbiology*.

[24] Beighton D, Ludford R, Clark DT (1995). Use of CHROMagar *Candida* medium for isolation of yeasts from dental samples. *Journal of Clinical Microbiology*.

[25] Rousselle P, Freydiere AM, Couillerot PJ, De Montclos H, Gille Y (1994). Rapid identification of *Candida albicans* by using albicans ID and fluoroplate agar plates. *Journal of Clinical Microbiology*.

[26] Pfaller MA, Houston A, Coffmann S (1996). Application of CHROMagar *Candida* for rapid screening of clinical specimens for *Candida albicans*, *Candida tropicalis*, *Candida krusei*, and *Candida (Torulopsis) glabrata*. *Journal of Clinical Microbiology*.

[27] Sullivan DJ, Westerneng TJ, Haynes KA, Bennett DE, Coleman DC (1995). *Candida dubliniensis* sp. nov.: phenotypic and molecular characterization of a novel species associated with oral candidosis in HIV-infected individuals. *Microbiology*.

[28] Schoofs A, Odds FC, Colebunders R, Ieven M, Goossens H (1997). Use of specialised isolation media for recognition and identification of *Candida dubliniensis* isolates from HIV-infected patients. *European Journal of Clinical Microbiology and Infectious Diseases*.

[29] Pinjon E, Sullivan D, Salkin I, Shanley D, Coleman D (1998). Simple, inexpensive, reliable method for differentiation of *Candida dubliniensis* from *Candida albicans*. *Journal of Clinical Microbiology*.

[30] Ellepola ANB, Morrison CJ (2005). Laboratory diagnosis of invasive candidiasis. *The Journal of Microbiology*.

[31] Aubert D, Puygauthier-Toubas D, Leon P (1996). Characterization of specific anti-Candida IgM, IgA and IgE: diagnostic value in deep-seated infections. *Mycoses*.

[32] Wahyuningsih R, Freisleben HJ, Sonntag HG, Schnitzler P (2000). Simple and rapid detection of *Candida albicans* DNA in serum by PCR for diagnosis of invasive candidiasis. *Journal of Clinical Microbiology*.

[33] Yeo SF, Wong B (2002). Current status of nonculture methods for diagnosis of invasive fungal infections. *Clinical Microbiology Reviews*.

[34] Budtz-Jörgensen E (1990). Histopathology, immunology, and serology of oral yeast infections. Diagnosis of oral candidosis. *Acta Odontologica Scandinavica*.

[35] Shepard JR, Addison RM, Alexander BD (2008). Multicenter evaluation of the *Candida albicans*/*Candida glabrata* peptide nucleic acid fluorescent in situ hybridization method for simultaneous dual-color identification of C. albicans and C. glabrata directly from blood culture bottles. *Journal of Clinical Microbiology*.

[36] Trnovsky J, Merz W, Della-Latta P, Wu F, Arendrup MC, Stender H (2008). Rapid and accurate identification of *Candida albicans* isolates by use of PNA FISHFlow. *Journal of Clinical Microbiology*.

